# The Silent Fracture: Incidental CBCT Detection of a Non‐Traumatic Oblique Palatal Root Fracture in a Vital Maxillary Molar; A Case Report and a Mini Review

**DOI:** 10.1155/crid/4875588

**Published:** 2026-07-18

**Authors:** Mohsen Aminsobhani, Somayeh Majidi

**Affiliations:** ^1^ Dental School, AJA University of Medical Sciences, Tehran University of Medical Sciences, Tehran, Iran, tums.ac.ir; ^2^ Department of Endodontics, School of Dentistry, Tehran University of Medical Sciences, Tehran, Iran, tums.ac.ir

**Keywords:** asymptomatic fracture, cone-beam computed tomography, maxillary molar, nontraumatic etiology, oblique root fracture, persian population

## Abstract

Root fractures of maxillary molars, particularly those involving the palatal root, are rarely reported and often present diagnostic challenges due to their deep anatomical position and lack of overt clinical symptoms. Most cases are associated with trauma; however, nontraumatic etiologies, including chronic occlusal stress and dietary habits, may also contribute. A 57‐year‐old Iranian female with no history of trauma or systemic disease was referred for endodontic evaluation of the maxillary first molar due to extensive caries and a separated instrument fragment. Cone‐beam computed tomography (CBCT), performed for diagnostic clarification, incidentally revealed an oblique palatal root fracture in the adjacent intact maxillary second molar (Tooth #15). The fracture line extended obliquely from the furcation area toward the apical third of the palatal root. The tooth remained vital, asymptomatic, and clinically stable, with normal probing depths and intact surrounding soft tissues. Conservative management through observation and periodic radiographic monitoring was adopted. At the 8‐month follow‐up, the tooth remained asymptomatic and responsive to vitality testing, with no clinical or radiographic evidence of periodontal or periapical pathology. This case highlights the diagnostic value of CBCT in identifying asymptomatic root fractures that may remain undetected on conventional radiographs. It expands the etiological understanding of root fractures to include repetitive masticatory microstresses potentially associated with dietary habits such as the consumption of hard traditional foods (e.g., *Tah dig*). Accurate diagnosis requires clinical expertise, careful examination, and the use of advanced imaging modalities. To the best of our knowledge, this is the first report of an incidentally detected asymptomatic oblique palatal root fracture in an Iranian patient. The case underscores the importance of clinician awareness, comprehensive imaging assessment, and conservative management for preserving pulp vitality and long‐term tooth function.

## 1. Introduction

Root fractures are relatively uncommon dental injuries, most frequently associated with acute trauma and predominantly affecting anterior teeth [1–3]. In contrast, nontraumatic root fractures in posterior teeth—particularly in vital, nonendodontically treated molars—are exceedingly rare and often under‐recognized in clinical practice. These fractures may develop insidiously and remain asymptomatic for extended periods, contributing to their underdiagnosis [1–4]

Unlike trauma‐induced fractures, nontraumatic root fractures are generally attributed to chronic mechanical loading and fatigue‐related mechanisms. Age‐related changes in dentin, including reduced elasticity and increased brittleness, combined with cumulative occlusal stress, play a significant role in their development. Epidemiological studies have shown that such fractures are more prevalent in older individuals, typically between the fifth and seventh decades of life, and are often associated with posterior teeth exhibiting significant occlusal wear [3, 5]. In a large cross‐sectional study, the incidence of fatigue‐related root fractures in nonendodontically treated teeth was reported to be less than 1%, highlighting their rarity [6].

Anatomical and biomechanical factors further contribute to fracture susceptibility. Roots with elongated or oval cross‐sectional morphology demonstrate reduced resistance to cyclic loading, increasing the risk of crack initiation and propagation [2, 6]. Additionally, uneven occlusal wear patterns may alter stress distribution within the root structure, concentrating forces in critical regions such as the middle third of the root and predisposing the tooth to fracture [7]

Dietary habits have also been implicated as contributing factors. Frequent consumption of hard or fibrous foods—such as nuts, bone fragments, or culturally specific items like *Tah dig* (a traditional Iranian dish characterized by a hardened, crispy layer formed during cooking)—can subject teeth to repetitive occlusal loading and accelerate dentin fatigue over time [7–9]. These repetitive micro stresses may eventually lead to the formation of microcracks and progression to complete root fractures, even in intact, unrestored teeth.

Among posterior teeth, maxillary molars—particularly the palatal root—are of special interest due to their anatomical complexity and load‐bearing function. Fractures in this region are especially difficult to detect because of their deep location, superimposition of surrounding structures, and frequent absence of clinical signs such as pain, mobility, or periodontal defects [10, 11]. As a result, such fractures are often discovered incidentally during radiographic examinations performed for unrelated reasons.

From a diagnostic perspective, conventional two‐dimensional radiography has significant limitations in detecting root fractures, with only a minority of fracture lines being directly visualized [12]. Early‐stage fractures may not present with radiographic or clinical signs, further complicating diagnosis. In this context, cone‐beam computed tomography (CBCT) has emerged as a valuable tool, providing three‐dimensional visualization of fracture lines, assessment of fragment separation, and evaluation of surrounding periodontal structures with greater accuracy [13–16].

The clinical relevance of identifying asymptomatic root fractures lies in preventing potential long‐term complications, including pulp necrosis, periodontal breakdown, and challenges in restorative or implant treatment planning [1, 11, 17]. Early detection allows for appropriate monitoring and conservative management, preserving tooth vitality and function

This report presents a rare case of an incidentally detected, nontraumatic oblique palatal root fracture in a vital maxillary second molar in a Persian patient. The case highlights the diagnostic value of CBCT, the potential role of chronic occlusal and dietary factors in fracture development, and the importance of clinician awareness in identifying these silent yet clinically significant conditions.

## 2. Materials and Methods

This case report was prepared in accordance with the CARE (CAse REport) guidelines to ensure completeness, transparency, and standardization of reporting. Additionally, elements of the PRICE 2020 (Preferred Reporting Items for Case Reports in Endodontics) guidelines were considered to enhance endodontic‐specific reporting

Written informed consent was obtained from the patient for both clinical management and publication of anonymized clinical and radiographic data.

The case was selected based on the incidental radiographic detection of an oblique root fracture involving the palatal root of a maxillary second molar. The tooth was clinically asymptomatic, vital at the time of diagnosis

The inclusion criteria for reporting were as follows:

1. absence of a history of dental trauma;
2. confirmation of the root fracture using CBCT;
3. maintenance of pulp vitality and normal periodontal parameters; and
4. availability of comprehensive clinical and radiographic documentation

A completed CARE checklist is provided as supporting information (Available here).

## 3. Case Presentation

### 3.1. Patient Information

A 57‐year‐old Iranian female patient presented to the dental clinic for routine evaluation and management of dental caries. The patient′s medical history was noncontributory, with no reported systemic conditions or medication use. There was no history of dental trauma or previous endodontic treatment in the maxillary second molar.

### 3.2. Clinical Findings

Clinical examination revealed a carious lesion in the maxillary first molar, as shown in the intraoral photograph (Figure 1, B‐C). The adjacent maxillary second molar appeared clinically intact, with no visible restorations, cracks, or discoloration.

**Figure 1:**
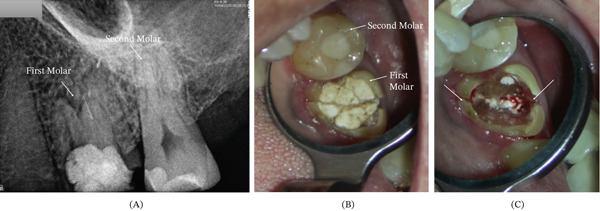
Preoperative radiograph (A) and intraoral photographs (B‐C) of the maxillary first and second molars. The carious lesion is indicated by white arrows in the right photograph.

Periodontal examination demonstrated normal probing depths and no evidence of attachment loss. The maxillary second molar responded positively to pulp vitality testing, indicating a vital pulp. There was no tenderness to percussion or palpation, and no abnormal mobility was detected. The surrounding soft tissues appeared healthy, with no signs of inflammation, swelling, or sinus tract formation.

A preoperative periapical radiograph (Figure 1A, left) did not reveal any obvious abnormalities in the maxillary second molar.

### 3.3. Timeline

The chronological sequence of diagnostic and follow‐up procedures is summarized in Table 1.

**Table 1:** Chronological timeline of diagnostic procedures, clinical findings, treatment decisions, and follow‐up assessments related to the incidental detection and conservative management of the oblique palatal root fracture.

Time	Clinical event
Initial visit	Clinical examination and periapical radiograph (Figure 1)
Same visit	CBCT imaging performed (Figure 2)
Diagnosis	Incidental detection of palatal root fracture
Treatment	Caries management of adjacent tooth; conservative monitoring (Figures 3)
Follow‐up	Clinical and intraoral evaluation (Figures 4 and 5)

### 3.4. Diagnostic Assessment

Although the initial periapical radiograph did not demonstrate any signs of root fracture (Figure 1A, left), further evaluation using CBCT was performed.

CBCT imaging revealed a distinct oblique fracture line involving the palatal root of the maxillary second molar. The fracture was most clearly visualized in the coronal section (Figure 2A), where separation of the root fragments could be identified. Additional CBCT views (Figure 2B–C) confirmed the extent and orientation of the fracture without evidence of associated periapical pathology.

**Figure 2:**
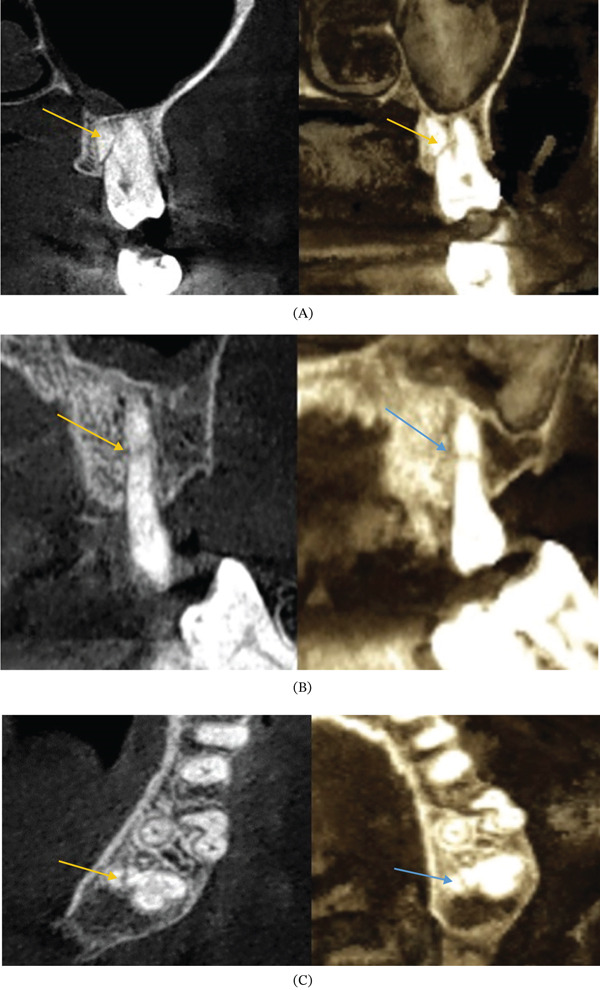
CBCT images of the maxillary second molar demonstrating an oblique fracture of the palatal root. (A) Coronal and (B) sagittal views illustrate the fracture line extending from the furcation toward the apical segment, whereas the (C) axial view reveals the mesiodistal orientation of the palatal root fracture, indicated by arrows.

These findings were further illustrated schematically (Figure 3), demonstrating the trajectory of the fracture line from the cervical region toward the apical third of the palatal root.

**Figure 3:**
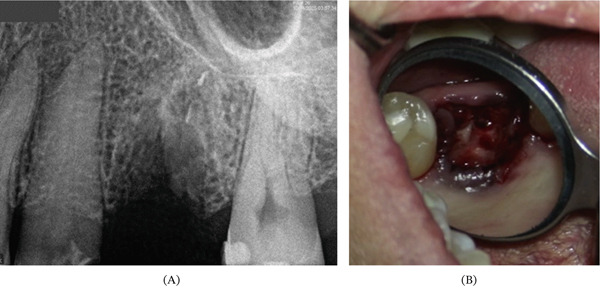
Periapical radiograph (A) and photograph (B) demonstrating extraction of Ttooth #14 due to extensive carious involvement and the presence of a separated endodontic instrument within the canal system. Notably, no radiographic evidence of root fracture is observed in the adjacent maxillary second molar (tTooth #15).

Differential diagnoses included cracked tooth syndrome and vertical root fracture; however, the absence of clinical symptoms and the characteristic oblique fracture pattern observed on CBCT supported the diagnosis of a nontraumatic horizontal root fracture (HRF).

### 3.5. Therapeutic Intervention

Given the absence of symptoms, preservation of pulp vitality, and lack of periodontal involvement, a conservative management approach was adopted. No endodontic or surgical intervention was indicated.

The patient received treatment for the carious lesion in the adjacent maxillary first molar (Figure 1) and was advised to avoid excessive occlusal loading on the affected tooth. Dietary counseling was provided, particularly regarding the reduction of hard food consumption.

### 3.6. Follow‐Up and Outcomes

At follow‐up evaluation, the maxillary second molar remained asymptomatic, with preserved pulp vitality and no evidence of mobility or periodontal deterioration.

Follow‐up intraoral examination (Figure 4) demonstrated healthy surrounding soft tissues, with no signs of inflammation or swelling adjacent to the fracture site. The tooth remained functionally stable, supporting continued conservative monitoring.

**Figure 4:**
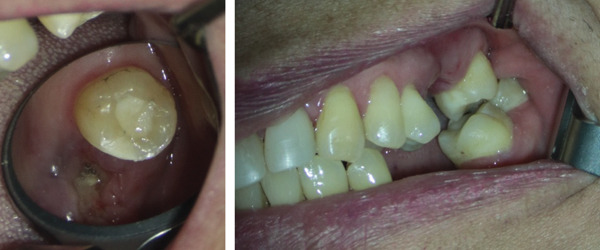
Follow‐up intraoral photograph of the maxillary second molar demonstrating the absence of soft‐tissue inflammation or swelling adjacent to the fracture line.

At the [8‐]month follow‐up, a comprehensive clinical and radiographic assessment was performed. The maxillary second molar remained free of signs and symptoms and responded positively to cold vitality testing, confirming continued pulp vitality. No tenderness to percussion or palpation was detected. The adjacent intraoral soft tissues appeared completely healed, with no evidence of swelling or inflammation, as illustrated in Figure 5A.

**Figure 5:**
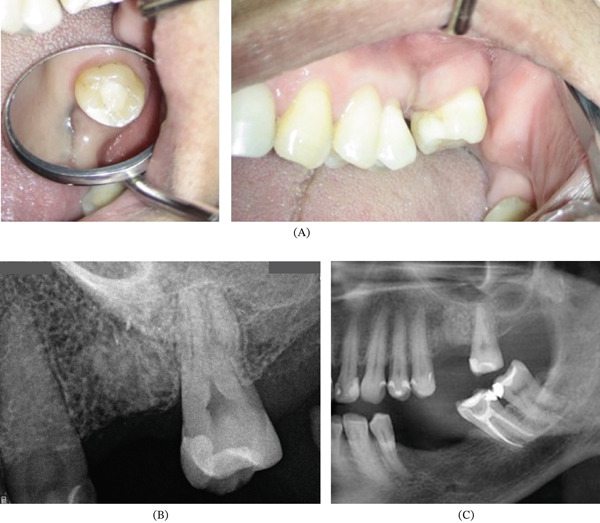
Eight‐month follow‐up evaluation of the maxillary second molar. (A) Intraoral photograph demonstrating complete healing of the adjacent soft tissues, with no evidence of swelling or inflammation. (B) Periapical radiograph showing absence of periapical or periodontal pathology and maintenance of normal periapical structures. (C) Panoramic segment confirming the absence of pathological changes associated with the affected tooth and preservation of surrounding supporting structures.

Radiographic evaluation (Figure 5B) revealed no signs of periapical pathology or periodontal involvement, and the periapical structures remained within normal limits. Additionally, a panoramic segment (Figure 5C) further confirmed the absence of pathological changes associated with the affected tooth.

The patient was advised to proceed with implant placement in the maxillary first molar region and in the posterior mandibular region to optimize occlusal distribution and reduce functional loading on the affected tooth. Continued periodic follow‐up and avoidance of excessive occlusal stress were also recommended.

## 4. Literature Review of Reported Cases

A review of reported cases of HRFs in molars without trauma or endodontic etiology reveals variable clinical presentations and diagnostic pathways. These cases range from symptomatic fractures presenting with pain and periodontal signs to asymptomatic ones discovered incidentally through advanced imaging such as CBCT. Fractures predominantly involve palatal roots but have also been identified in other molar roots (mesiobuccal and distobuccal roots) with variable clinical impact and prognoses.

The following table (Table 2) summarizes key case reports highlighting clinical symptoms, root location, racial background, detection methods, outcomes, and prognoses.

**Table 2:** Summary of reported horizontal root fracture cases (1994–2025).

Case reference (Ref.)	Year	Number of fractured teeth	Root location	Symptoms	Racial/ethnic background	Detection method	Outcome/prognosis	Etiology
Jerome [11]	1994	1	Maxillary first molar, mesiobuccal root	Not specified	Caucasian	Periapical radiograph, endodontic retreatment	Resolved with root amputation; favorable outcome at 2‐year follow‐up	Iatrogenic (sinus surgery)
Legan et al. [18]	1995	1	Maxillary second premolar (root not specified)	Not reported	Not specified	Periapical radiograph	Guarded prognosis; required root canal treatment and root amputation	Iatrogenic (crown seating force)
Borelli et al. [19]	1999	2	Maxillary molars (roots not specified)	No pain, no abscess	Not specified	Periapical radiograph	One tooth developed abscess after 9 years; the other remained asymptomatic for 11 years	Occlusal trauma, bruxism
Çobankara and ÜNGÖR [20]	2007	1	Maxillary first premolar, palatal root	Asymptomatic	Not specified	Routine radiograph ± CBCT	Tooth remained vital; favorable long‐term outcome	Spontaneous/nontraumatic fatigue fracture
Lin et al. [2]	2008	2	Palatal roots of maxillary molars	Periodontal destruction	Chinese	Radiographs, CBCT, surgical confirmation	Guarded prognosis if untreated	Chronic mechanical stress, infection
Wang et al. [10]	2011	6	Maxillary molars (various roots)	Variable symptoms	Chinese	Radiographs, CBCT	Mixed outcomes; some teeth required intervention	Trauma, occlusal forces
Clarkson et al. [17]	2015	1	Palatal root of maxillary first premolar	No pain; pulp vitality maintained	Not specified	Radiographs, CBCT	Favorable prognosis with conservative management	Nontraumatic fatigue fracture
Tsai et al. [1]	2017	41	Posterior teeth (various roots)	Pain, increased mobility	Taiwanese	Clinical examination, multi‐angulated radiographs, CBCT	Variable; prognosis dependent on early diagnosis	Trauma and attrition
Badar et al. [21]	2018	1	Palatal root of maxillary first premolar	Mild percussion pain	Not specified	Radiographs	Favorable healing following flexible splinting	Occlusal trauma (biting hard object)
Da Silva et al. [22]	2018	1	Mesial root of mandibular second molar	Asymptomatic	Not specified	Radiographs	Spontaneous healing	Iatrogenic trauma during surgery
Łagocka R, et al. [23]	2021	1	Mesial root of molar	Asymptomatic	Not specified	Incidentally detected on imaging	Favorable outcome with conservative monitoring	Nontraumatic incidental fracture
Present case (Aminsobhani and Majidi)	2026	1	Palatal root of maxillary second molar	Asymptomatic	Iranian	Incidental CBCT finding	Favorable outcome with conservative monitoring	Chronic occlusal microstress, dietary habits

## 5. Discussion

HRFs involving the palatal root of maxillary molars remain uncommon clinical findings and are frequently overlooked due to their subtle presentation and complex anatomical location [2, 20]. The present case is notable for the identification of an oblique fracture pattern in a vital, unrestored tooth, detected incidentally in the absence of clinical symptoms. Such cases highlight the existence of a subset of HRFs that deviate from the classical trauma‐related presentation and instead follow a more insidious course.

Although HRFs are traditionally associated with acute trauma in younger populations, this case supports the growing body of evidence indicating that nontraumatic fractures may occur in older individuals as a consequence of cumulative mechanical fatigue. Age‐related changes in dentin properties, including reduced elasticity and increased brittleness, may predispose teeth to structural compromise under repeated loading conditions. In this context, chronic occlusal forces appear to play a critical role in crack initiation and propagation over time [24].

An important contributing factor in the present case is the patient′s long‐term dietary habit of consuming hard foods (e.g., *Tah dig* is a traditional Iranian food consisting of a deliberately hardened, crispy rice, bread, or potato crust formed at the bottom of the cooking pot, which requires increased masticatory force during consumption and may contribute to repetitive occlusal microstress on posterior teeth over time). The repetitive masticatory loading required for foods such as *Tah dig* may generate sustained occlusal microstress, particularly in posterior teeth. This case demonstrates the potential for such dietary patterns to induce structural fatigue even in intact, vital teeth without prior restorative intervention. The gradual accumulation of microdamage may ultimately predispose the palatal root to fracture. This observation is consistent with reports suggesting that fatigue‐related HRFs are more prevalent in later decades of life [24].

A key clinical feature of this case is its asymptomatic presentation. The absence of pain, mobility, or periodontal involvement is consistent with previously reported nontraumatic HRFs and contributes to the diagnostic challenge. Without overt clinical signs, these fractures may remain undetected until identified incidentally or after the development of secondary complications [15, 25–27].

From a diagnostic standpoint, this case reinforces the limitations of conventional two‐dimensional radiography. The inability of periapical radiographs to reveal the fracture line in this case reflects the well‐documented issue of anatomical superimposition and unfavorable fracture orientation. In contrast, CBCT provided definitive visualization of the fracture morphology and fragment separation, particularly in the coronal plane (Figure 2A). This finding underscores the essential role of CBCT in cases where clinical suspicion persists despite inconclusive conventional imaging [15, 25].

The clinical findings in this case—namely preserved pulp vitality, absence of periodontal pathology, and lack of symptoms—supported a conservative management approach. Current evidence suggests that asymptomatic HRFs in vital teeth may be successfully managed through observation and periodic monitoring. Intervention should be limited to cases demonstrating pulpal or periodontal deterioration. When treatment becomes necessary, bioactive materials such as mineral trioxide aggregate (MTA) have shown favorable outcomes in maintaining periapical health [24, 27–29]. The decision to preserve the tooth in this case aligns with contemporary principles emphasizing minimally invasive and vitality‐preserving strategies [4, 5, 28]. Notably, the favorable findings observed during the 8‐month follow‐up period—including maintained pulp vitality, absence of clinical symptoms, and lack of radiographic pathology—further support the feasibility of conservative monitoring in carefully selected cases of nontraumatic HRFs. These findings suggest that immediate invasive intervention may not be necessary when clinical and radiographic stability can be maintained over time.

Another aspect of interest is the potential influence of racial and ethnic factors on the occurrence of HRFs. Previous studies have reported a higher prevalence of root fractures in East Asian populations, possibly related to genetic, anatomical, or dietary factors [16, 24]. However, data from Middle Eastern populations remain limited. To the best of our knowledge, this case represents one of the first documented instances of a nontraumatic HRF in a Persian patient. This observation highlights the importance of expanding the epidemiological database to include diverse populations and suggests that cultural factors, including dietary habits, may play a role in fracture risk [16, 24].

Importantly, this case emphasizes the role of clinical vigilance in detecting otherwise silent conditions. Accurate diagnosis of HRFs requires not only appropriate imaging modalities but also a high index of suspicion, particularly in patients presenting with risk factors such as advanced age or significant occlusal loading. The integration of clinical findings with targeted use of CBCT is essential for early detection and appropriate management [24, 30].

In summary, this case supports the concept that HRFs of the posterior maxilla may develop in the absence of trauma and may remain clinically silent. Chronic occlusal loading and dietary habits appear to contribute to fatigue‐related fracture mechanisms, whereas CBCT plays a pivotal role in diagnosis. The findings underscore the importance of conservative management in asymptomatic cases and contribute to the limited body of evidence on nontraumatic HRFs in diverse populations.

## 6. Limitations

This report describes a single clinical case, and therefore its findings should be interpreted with caution and cannot be generalized to a wider population. Although an association between dietary habits, chronic occlusal microstress, and the observed palatal root fracture is proposed, a direct causal relationship cannot be confirmed. In addition, prefracture imaging was not available; therefore, the exact timing of fracture initiation and progression could not be determined.

Although the tooth remained clinically and radiographically stable during the 8‐month follow‐up period, longer observation is still necessary to fully evaluate the long‐term prognosis and biological behavior of asymptomatic palatal root fractures managed conservatively. Furthermore, occlusal forces, parafunctional habits, and masticatory load were not objectively measured, and the suggested contribution of hard‐food consumption was based primarily on patient self‐report. Finally, because surgical intervention was not indicated, histological confirmation of the fracture and surrounding tissues was not possible, limiting microscopic evaluation of the defect.

## 7. Future Directions

This case underscores the need for further research into nontraumatic root fractures in posterior teeth, particularly those that remain asymptomatic and are detected incidentally. Larger case series and prospective studies are needed to better clarify prevalence, predisposing factors such as occlusal wear and dietary habits, and the natural course of these fractures. Long‐term follow‐up data would help define prognosis and establish clearer criteria for conservative versus interventional management. Future work should also focus on optimizing diagnostic strategies, including the selective use of low‐dose CBCT and emerging tools such as artificial intelligence–assisted image analysis, to improve early detection while minimizing radiation exposure. Finally, continued adoption of standardized reporting frameworks, such as PRICE 2020, will facilitate meaningful comparison between cases and contribute to the development of evidence‐based clinical guidelines.

## 8. Conclusion

This case highlights the importance of maintaining a high index of suspicion for root fractures, even in asymptomatic posterior teeth of older adults. The incidental discovery of an oblique palatal root fracture in a vital, symptom‐free maxillary molar emphasizes the diagnostic superiority of CBCT over conventional radiography. Comprehensive assessment—including evaluation of dietary habits and occlusal function—is essential for recognizing potential nontraumatic etiologies such as chronic masticatory stress.

The favorable clinical and radiographic findings observed during the 8‐month follow‐up period further support the feasibility of conservative, vitality‐preserving management in carefully selected asymptomatic cases. Regular clinical and radiographic monitoring remains essential when no signs of pulpal or periodontal pathology are present.

Furthermore, documenting this case in a Persian individual draws attention to the potential underrecognition of such fractures in non‐Asian populations and underscores the importance of ethnic and cultural considerations in dental epidemiology. Ultimately, an individualized and multidisciplinary approach may contribute to optimal patient outcomes and improve the current understanding of HRF susceptibility and diagnosis.

## Author Contributions

Mohsen Aminsobhani: conceptualization, data curation, methodology, supervision, visualization review and editing. Somayeh Majidi: data curation, methodology, project administration, writing—original draft, writing—review and editing.

## Funding

No funding was received for this manuscript.

## Disclosure

Ensuring the highest standards of clarity and readability, this manuscript underwent a thorough review and editing process supported by advanced artificial intelligence (AI) tools. The AI assistance focused solely on refining sentence structure, enhancing coherence, and improving overall language flow without altering the original scientific content or intent. Importantly, AI was not involved in the conception of the study, development of the main ideas, or interpretation of the results, and therefore is not considered an author of this work. This approach facilitated clearer communication of complex ideas and helped eliminate ambiguities, thereby making the manuscript more accessible and engaging for a diverse readership. The use of AI in this capacity serves as a valuable supplement to the authors′ expertise, ensuring the presentation of the research is both precise and polished. All authors have read and approved the final version of the manuscript. Corresponding Author/Manuscript Guarantor had full access to all of the data in this study and takes complete responsibility for the integrity of the data and the accuracy of the data analysis.

## Consent

Written informed consents were obtained from the patients to publish this case report in accordance with the journal’s patient consent policy.

## Conflicts of Interest

The authors declare no conflicts of interest.

## Patient Perspective

The patient was informed about the condition and the conservative management approach. She expressed understanding and satisfaction with the decision to preserve the tooth and appreciated the importance of regular follow‐up visits.

## Supporting information


**Supporting Information** Additional supporting information can be found online in the Supporting Information section. Supporting Information. The CARE checklist for this case report is provided as supporting information and is available to support adherence to CARE reporting guidelines.

## Data Availability

The authors confirm that the data supporting the findings of this study are available within the article and/or its supporting information.
