# Supplementary material for: The Silent Fracture: Incidental CBCT Detection of a Non‐Traumatic Oblique Palatal Root Fracture in a Vital Maxillary Molar; A Case Report and a Mini Review
Source: Case Rep Dent. 2026 Jul 18;2026:4875588. doi: 10.1155/crid/4875588 (PMC13380034; doi:10.1155/crid/4875588)
Supplement: Supplementary file 1 — Supporting Information Additional supporting information can be found online in the Supporting Information section. Supporting Information. The CARE checklist for this case report is provided as supporting information and is available to support adherence to CARE reporting guidelines. [file CRID-2026-4875588-s001.docx]

**CARE Checklist**

| **No.** | **Item** | **Description** | **Reported (Yes/No)** | **Location in Manuscript** |
| --- | --- | --- | --- | --- |
| 1 | Title | The words “case report” are included in the title | Yes | Title page |
| 2 | Keywords | 2–5 relevant keywords provided | Yes | Abstract |
| 3 | Abstract | Structured summary including background, case, and conclusion | Yes | Abstract |
| 4 | Introduction | Background and rationale for the case | Yes | Section 1 |
| 5 | Patient Information | Demographics, medical and dental history | Yes | Section 3.1 |
| 6 | Clinical Findings | Symptoms and clinical examination findings | Yes | Section 3.2 |
| 7 | Timeline | Chronological description of events | Yes | Section 3.3 |
| 8 | Diagnostic Assessment | Diagnostic methods and reasoning | Yes | Section 3.4 |
| 9 | Therapeutic Intervention | Treatment procedures and rationale | Yes | Section 3.5 |
| 10 | Follow-up and Outcomes | Clinical course and outcomes | Yes | Section 3.6 |
| 11 | Discussion | Interpretation of findings and comparison with literature | Yes | Section 5 |
| 12 | Patient Perspective | Patient-reported experience or feedback | Yes | Section 8 |
| 13 | Informed Consent | Confirmation of written informed consent | Yes | End of manuscript |

| **No.** | **Item** | **Description** | **Reported (Yes/No)** | **Location in Manuscript** |
| --- | --- | --- | --- | --- |
| **1** | **Title** | **The words “case report” are included in the title** | **Yes** | **Title page** |
| **2** | **Keywords** | **2–5 relevant keywords provided** | **Yes** | **Abstract** |
| **3** | **Abstract** | **Structured summary including background, case, and conclusion** | **Yes** | **Abstract** |
| **4** | **Introduction** | **Background and rationale for the case** | **Yes** | **Section 1** |
| **5** | **Patient Information** | **Demographics, medical and dental history** | **Yes** | **Section 3.1** |
| **6** | **Clinical Findings** | **Symptoms and clinical examination findings** | **Yes** | **Section 3.2** |
| **7** | **Timeline** | **Chronological description of events** | **Yes** | **Section 3.3** |
| **8** | **Diagnostic Assessment** | **Diagnostic methods and reasoning** | **Yes** | **Section 3.4** |
| **9** | **Therapeutic Intervention** | **Treatment procedures and rationale** | **Yes** | **Section 3.5** |
| **10** | **Follow-up and Outcomes** | **Clinical course, 8-month follow-up findings, and outcomes** | **Yes** | **Section 3.6** |
| **11** | **Discussion** | **Interpretation of findings and comparison with literature** | **Yes** | **Section 5** |
| **12** | **Patient Perspective** | **Patient-reported experience or feedback** | **Yes** | **Section 8** |
| **13** | **Informed Consent** | **Confirmation of written informed consent** | **Yes** | **End of manuscript** |
